# Molecular determinants of prognosis in oesophageal cancer patients treated with chemoradiation

**DOI:** 10.1038/sj.bjc.6604627

**Published:** 2008-09-02

**Authors:** J A Ajani

**Affiliations:** 1 Department of GI Medical Oncology, University of Texas MD Anderson Cancer Center, Gastroesophageal Multidisciplinary Translation Research, Houston, TX 77030, USA

**Sir,**

The paper by Yoshikawa *et al* (2008) is of considerable interest to our group. The authors assessed the expression of Gli-1 in the residual squamous cell carcinoma of the oesophagus in the resected specimens of the patients treated with preoperative chemoradiation. The expression of Gli-1 correlated with poor prognosis. When employing the strategy of preoperative chemoradiation in oesophageal carcinoma, the prognosis of the patients can be determined by assessing the degree of residual cancer (Chirieac *et al*, 2005; Wu *et al*, 2007). In addition, the residual cancer correlates with metastatic potential (Rohatgi *et al*, 2005). It would be of interest to document that Gli-1 expression has an independent prognostic value. The authors did not report a multivariate analysis. Our group first documented the expression and temporal kinetics of sonic hedgehog ligand and Gli-1 protein expression in a xenograft model using oesophageal cancer cell lines (Sims-Mourtada *et al*, 2006). Both biomarkers correlated with chemoradiation resistance and dynamics of tumour repopulation after chemoradiation (Sims-Mourtada *et al*, 2006). Furthermore, our group documented that sonic hedgehog signalling mediates chemotherapy resistance through multiple drug transporter mechanisms and that this resistance can be overcome by inhibitors of the sonic hedgehog pathway (Sims-Mourtada *et al*, 2007). Unfortunately, Yoshikawa *et al* (2008) have neglected to discuss these two publications.
